# Microvascular inflammation is a risk factor in kidney transplant recipients with very late conversion from calcineurin inhibitor-based regimens to belatacept

**DOI:** 10.1186/s12882-020-01992-6

**Published:** 2020-08-20

**Authors:** Mira Choi, Friederike Bachmann, Kaiyin Wu, Nils Lachmann, Danilo Schmidt, Susanne Brakemeier, Michael Duerr, Andreas Kahl, Kai-Uwe Eckardt, Klemens Budde, Peter Nickel

**Affiliations:** 1 Department of Nephrology and Medical Intensive Care, Charité Universitätsmedizin Berlin, Augustenburger Platz 1, 13353 Berlin, Germany; 2 Department of Pathology, Charité Universitätsmedizin Berlin, Berlin, Germany; 3 Tissue Typing Laboratory, Charité Universitätsmedizin Berlin, Berlin, Germany

**Keywords:** Belatacept, Allograft failure, Kidney transplantation, Calcineurin inhibitor toxicity

## Abstract

**Background:**

In de novo kidney transplant recipients (KTR) treatment with belatacept has been established as a comparable option as maintenance immunosuppression, preferably as a strategy to convert from calcineurin inhibitor (CNI)- to belatacept-based immunosuppression. Switch to belatacept demonstrated improved renal function in patients with CNI-induced nephrotoxicity, but risk of transplant rejection and the development of donor-specific antibodies (DSA) are still a matter of debate. Only few data are available in patients at increased immunological risk and late after transplantation.

**Methods:**

We analyzed 30 long-term KTR (including 2 combined pancreas-KTR) converted from CNI to belatacept > 60 months after transplantation with moderate to severe graft dysfunction (GFR ≤ 45 mL/min). Biopsies were classified according to the Banff 2015 criteria. Group differences were assessed in a univariate analysis using Mann Whitney U or Chi square test, respectively. Multivariate analysis of risk factors for treatment failure was performed using a binary logistic regression model including significant predictors from univariate analysis. Fifty-six KTR matched for donor and recipient characteristics were used as a control cohort remaining under CNI-treatment.

**Results:**

Patient survival in belatacept cohort at 12/24 months was 96.7%/90%, overall graft survival was 76.7 and 60.0%, while graft survival censored for death was 79.3%/66.7%. In patients with functioning grafts, median GFR improved from 22.5 mL/min to 24.5 mL/min at 24 months. Positivity for DSA at conversion was 46.7%. From univariate analysis of risk factors for graft loss, GFR < 25 mL/min (*p* = 0.042) and Banff microvascular inflammation (MVI) sum score ≥ 2 (*p* = 0.023) at conversion were significant at 24 months. In the analysis of risk factors for treatment failure, a MVI sum score ≥ 2 was significant univariately (*p* = 0.023) and in a bivariate (*p* = 0.037) logistic regression at 12 months. DSA-positivity was neither associated with graft loss nor treatment failure. The control cohort had comparable graft survival outcomes at 24 months, albeit without increase of mean GFR in patients with functioning grafts (ΔGFR of − 3.6 ± 8.5 mL/min).

**Conclusion:**

Rescue therapy with conversion to belatacept is feasible in patients with worsening renal function, even many years after transplantation. The benefit in patients with MVI and severe GFR impairment remains to be investigated.

## Background

Long-term calcineurin inhibitor (CNI) exposure has been associated with numerous adverse effects such as nephrotoxicity, infections, hypertension, diabetes and dyslipidemia [1, 2]. Belatacept is a selective co-stimulation inhibitor that has been developed for CNI-free treatment. In de novo kidney transplant recipients treatment with belatacept has shown superior graft function and better patient and/or graft survival until 7 years post-transplant compared to cyclosporine despite increased early acute rejection rates [3–5]. Furthermore, belatacept use was associated with lower frequencies of patients developing donor-specific antibodies (DSA) [6]. While increased acute cellular rejection rates point to a lesser efficacy of belatacept compared to CNI, reasons for the observed lower DSA rates might be an improved adherence to the intravenous belatacept regimen or superior B-cell control under belatacept. In fact, some studies suggested that belatacept may have regulatory effects on B cells [7, 8].

Several studies have evaluated risks and benefits of conversion from CNI-based treatment to belatacept [9–12]. However, few data are available on late (> 60 months post-transplant) conversion of patients with high immunological risk factors, such as DSA, after transplantation [13–16].

Here, we analyzed a cohort of kidney and combined kidney and pancreas transplant recipients at our center converted from CNI-based regimens to belatacept at a very late time point of > 60 months after transplantation for predictive factors for graft loss or GFR deterioration after a period of 12 and 24 months. Patients were at increased immunological risk with a high percentage of 47% DSA-positivity at the time of conversion.

## Methods

This retrospective study analyzed the outcome in all adult kidney and combined kidney and pancreas transplant patients with moderate to severe graft dysfunction (GFR ≤ 45 mL/min) that were converted from a CNI-based maintenance immunosuppressive regimen to belatacept at a very late stage of > 60 months after last transplantation between 11/2012 and 11/2016. Chronic Kidney Disease Epidemiology Collaboration (CKD-EPI) formula was used to calculate estimate glomerular filtration rates. All patients were Epstein-Barr seropositive. At the time of conversion, belatacept was administered at a dose of 5 mg/kg intravenously on days 1, 15, 29, 43 and 57 in line with Rostaing et al. [12]. Thereafter, treatment was continued every 4 weeks. Due to CNI toxicity after long-term transplantation calcineurin inhibitor dose was tapered in a modified way as follows: to 50% the day after conversion (day 2), to 25% on day 15 and 0 on day 29 and thereafter. Immunosuppressive co-medication was continued. Biopsies were classified according to the Banff 2015 criteria [17]. Biopsies taken before publication of the Banff 2015 classification were scored retrospectively.

The primary outcome was treatment failure defined as renal graft loss or deterioration of GFR at 12 and 24 months compared to baseline GFR at the time of conversion. Treatment success was defined as stable GFR or improvement at 12 and 24 months. The following risk factors were analyzed for association with treatment failure at 12 and 24 months: biopsy scores, gender, donor age, living donation, patient age, conversion time after transplantation, body mass index (BMI), post-transplant diabetes mellitus, systolic and diastolic blood pressure, immunosuppressive regimen, history of any rejection prior to conversion, DSA positivity, eGFR and proteinuria at the time of conversion.

### Matched-pair analysis

In order to generate a matched control group, a cohort of potential patients was identified using our web-based electronic patient record system “TBase” [18] in analogy to the belatacept treatment cohort. Patients were 1:2 matched for age (± 5 years), donor age (± 5 years), gender, immunosuppressive regimen with exclusion of mTOR therapy, GFR +/− 5 mL/min, GFR ≤ 45 between 2012 and 2016 and at start of observation period, and availability of renal transplant biopsies prior to observation.

### Data analysis

IBM SPSS statistics version 25.0 was used for statistical analysis. Group differences were assessed in a univariate analysis using Mann Whitney U (MWU) or Chi square test, respectively. Multivariate analysis of risk factors for treatment failure was performed using a binary logistic regression model including significant predictors from the univariate analysis.

## Results

A total of 30 patients with belatacept conversion from a CNI-based immunosuppressive regimen at a median time of 127.5 ± 91.3 (range 99–190) months after transplantation were included. Table 1 shows patient and graft survival at 12 and 24 months after conversion to belatacept. Clinical characteristics at baseline and by status of graft failure or treatment failure, at 12 and 24 months are shown in Tables 2 and 3, respectively.

**Table 1:** Patient survival, graft survival and renal function with and without imputation for missing values at 12 and 24 months after conversion to belatacept. GFR 9 mL/min was imputed for kidney graft loss

	Switch to belatacept (*N* = 30) GFR at switch = 22.5 ± 12 mL/min		Control cohort (*N* = 56) GFR at start of observation = 24.5 ± 14 mL/min	
Timepoint after switch	12 months	24 months	12 months	24 months
Patient survival	96.7% (29/30)	90% (27/30)	96.4% (54/56)	91.07% (51/56)
Death with functioning graft	1/30	3/30	2/56	5/56
Kidney or pancreas graft loss	6/30	9/30	8/56	16/56
Death-censored kidney graft survival	79.3% (23/29)	66.7% (18/27)	85.2% (46/54)	68.6% (35/51)
Overall kidney graft survival	76.7% (23/30)	60.0% (18/30)	82.1% (46/56)	62.5% (35/56)
Median GFR in patients without graft loss	23.3 ± 15	24.5 ± 15.0	23.0 ± 15	24.0 ± 9
ΔGFR from GFR at baseline in patients without graft loss	1.3 ± 5.9	1.8 ± 7.9	−2.0 ± 7.2	− 3.5 ± 8.6
Median GFR with imputation for graft loss	21.5 ± 18	18.5 ± 21	21.5 ± 18	19 ± 17
ΔGFR from GFR at baseline in patients with imputation for graft loss	−0.56 ± 6.8	− 1.54 ± 8.4	−3.3 ± 7.6	−5.5 ± 8.3

Data were expressed as medians (interquartile range), means (standard deviation) or numbers

GFR glomerular filtration rate

**Table 2:** Baseline characteristics in belatacept cohort by status for graft failure at 12 and 24 months censored for death

Patient characteristics	All patients (*N* = 30)	Functioning graft at 12 months (*N* = 23)	Graft failure at 12 months (*N* = 6)	*P* value	Functioning graft at 24 months (*N* = 18)	Graft failure at 24 months (*N* = 9)	*P* value
Age (y)	53.5 ± 26	54.0 ± 19	43 ± 23	0.302	51.5 ± 23	48 ± 28	0.940
Donor age (y)	48.0 ± 23	50.0 ± 26	44 ± 19	0.555	45 ± 26	48.5 ± 23	0.825
Gender (m/f)	20/10	15/8	4/2	1.000	12/6	5/4	0.683
Post-transplant diabetes	3/30	3/23	0/6	1.000	3/18	0/9	0.529
BMI	25.3 ± 4.1	24.9 ± 3.9	25.4 ± 6.6	0.511	24.1 ± 5.6	25.5 ± 5.6	0.194
Systolic BP (mmHg)	134 ± 17	138 ± 20	131.5 ± 30	0.384	136.5 ± 21	133 ± 16	0.348
Diastolic BP (mmHg)	84 ± 10	84 ± 10	84 ± 31	0.581	86 ± 11	84 ± 18	0.691
Time after transplantation (m)	127.5 ± 91.3	128 ± 130	127.5 ± 52.5	0.773	133.5 ± 135.5	126 ± 35	0.275
eGFR (mL/min)	22.5 ± 12	24.0 ± 13	19.0 ± 10	0.302	25.5 ± 12	18.0 ± 4	0.095
eGFR < 25 mL/min	17/30	12/23	5/6	0.354	8/18	8/9	**0.042**
Proteinuria (mg/g creatinine)	840 ± 1166	830 ± 950	1256 ± 3211	0.302	657 ± 1276	869 ± 2017	0.668
Living donor transplants	8/30	6/23	2/6	1.000	4/18	3/9	0.653
pancreas/kidney	2/30	–	–				
Immunosuppression							
Tacrolimus	22/30	15/23	6/6	0.148	13/18	6/9	1.000
Cyclosporine A	8/30	8/23	0/6	0.148	5/18	3/9	1.000
Mycophenolic acid	27/30	20/23	6/6	1.000	16/18	9/9	0.538
Azathioprin	2/30	2/23	0/6	1.000	1/18	0/9	1.000
Steroid	24/30	17/23	6/6	0.295	13/18	8/9	0.628
DSA	14/30	11/23	2/6	0.663	8/18	4/9	1.000
h/o any rejection	15/30	10/23	5/6	0.169	7/18	7/9	0.103
aTCMR	3/30	1/23	2/6	0.100	1/18	2/9	0.250
aABMR	6/30	4/23	2/6	0.575	2/18	4/9	0.136
Biopsy scores							
glomerular scarring (%)	30.5 ± 33	27 ± 31	42 ± 27	0.302	29 ± 27	32 ± 43	0.860
cg	0.0 ± 3.0	0.0 ± 3.0	1.25 ± 3.0	0.694	0.0 ± 1.1	2.5 ± 3.0	0.232
ct	1.0 ± 1.0	1.0 ± 1.0	1.0 ± 2.0	0.546	1.0 ± 1.0	1.0 ± 1.0	0.348
ci	1.0 ± 1.0	1.0 ± 1.0	1.0 ± 2.0	0.477	1.0 ± 1.0	1.0 ± 1.0	0.253
cv	2.0 ± 2.0	2.0 ± 2.0	1.5 ± 1.0	0.427	2.0 ± 3.0	2.0 ± 1.0	0.560
mm	1.0 ± 2.0	1.0 ± 1.0	2.0 ± 1.5	0.071	0.0 ± 1.0	2.0 ± 1.5	0.067
ah	3.0 ± 0.0	3.0 ± 0.0	3.0 ± 0.3	0.979	0.0 ± 1.0	3.0 ± 1.0	0.860
g	0.0 ± 1.0	0.0 ± 1.0	0.0 ± 1.9	0.694	0.0 ± 1.0	0.0 ± 1.8	0.668
t	0.0 ± 0.0	0.0 ± 0.0	0.0 ± 2.0	0.384	0.0 ± 0.0	0.0 ± 1.0	0.322
i	0.0 ± 0.0	0.0 ± 0.0	0.0 ± 2.0	0.546	0.0 ± 0.0	0.0 ± 1.0	0.781
v	0.0 ± 0.0	0.0 ± 0.0	0.0 ± 0.0	1.000	0.0 ± 0.0	0.0 ± 0.0	1.000
ptc	0.0 ± 0.0	0.0 ± 0.0	0.5 ± 3.0	0.102	0.0 ± 0.0	0.0 ± 0.0	0.067
C4d	0.0 ± 0.0	0.0 ± 0.0	0.0 ± 1.0	0.655	0.0 ± 0.0	0.0 ± 0.0	0.820
MVI sum score	0.0 ± 1.3	0.0 ± 1.0	1.25 ± 3.8	0.254	0.0 ± 0.0	2.0 ± 3.0	0.095
MVI sum score ≧2	7/30	4/23	3/6	0.131	2/18	5/9	**0.023**

Data were expressed as medians (interquartile range), or numbers

*BMI* body mass index, *BP* Blood pressure, *eGFR* estimated glomerular filtration rate, *DSA* donor specific antibodies, *h/o* history of, *aTCMR* active T cell mediated rejection, *aABMR* active antibody-mediated rejection, *MVI* microvascular inflammation

**Table 3:** Baseline characteristics in belatacept cohort by status for treatment success or failure censored for death. Treatment failure was defined as graft failure or GFR deterioration compared to the time of conversion

Patient characteristics	All patients (*N* = 30)	Treatment success at 12 months (*N* = 16)	Treatment failure at 12 months (*N* = 13)	*P* value	Treatment success at 24 months (*N* = 12)	Treatment failure at 24 months (*N* = 15)	*P* value
Age (y)	53.5 ± 26	53.5 ± 26	50 ± 29	0.983	52 ± 18	50 ± 30	0.829
Donor age (y)	48 ± 23	46.5 ± 31	46 ± 22	0.689	43 ± 26	52.5 ± 23	0.134
Gender (m/f)	20/10	11/5	8/5	0.714	10/2	7/8	0.107
Post-transplant diabetes	3/30	2/16	1/13	1.000	1/12	2/15	1.000
BMI	25.3 ± 4.1	24.7 ± 4.0	25.5 ± 6.1	0.650	24.7 ± 4.0	25.3 ± 7.9	0.100
Systolic BP (mmHg)	134 ± 17	139 ± 24	133 ± 18	0.199	139 ± 27	132 ± 14	0.183
Diastolic BP (mmHg)	84 ± 10	85 ± 10	84 ± 12	0.812	88.5 ± 12	83 ± 13	0.300
Time after transplantation (m)	127.5 ± 91.3	128.5 ± 148.8	127 ± 60	0.619	133.5 ± 125.8	126 ± 74	0.300
eGFR (mL/min)	22.5 ± 12	25 ± 12	20 ± 10	0.449	25.5 ± 14	20.0 ± 12	0.126
eGFR < 25 mL/min	17/30	7/16	10/13	0.130	5/12	11/15	0.130
Proteinuria (mg/g creatinine)	840 ± 1166	647 ± 1125	890 ± 1857	0.398	452 ± 1125	869 ± 1592	0.399
Living donor transplants	8/30	3/16	5/13	0.406	3/12	4/15	1.000
pancreas/kidney	2/30	–	–	–			
Immunosuppression							
Tacrolimus	22/30	12/4	9/4	1.000	9/12	10/15	0.696
Cyclosporine A	8/30	4/12	4/9	1.000	3/12	5/15	0.696
Mycophenolic acid	27/30	13/3	13/0	0.232	10/12	15/15	0.188
Azathioprin	2/30	2/14	0/13	0.488	1/12	0/15	0.444
Steroid	24/30	13/3	10/3	1.000	10/12	11/15	0.662
DSA	14/30	7/16	6/13	1.000	5/12	7/15	1.000
h/o any rejection	15/30	6/16	9/13	0.139	6/12	8/15	1.000
aTCMR	3/30	1/16	2/13	0.573	1/12	2/15	1.000
aABMR	6/30	1/16	5/13	0.064	1/12	5/15	0.182
Biopsy scores							
Glomerular scarring (%)	30.5 ± 33	26 ± 29	32 ± 35.5	0.423	27 ± 33	32 ± 36	0.829
Cg	0.0 ± 3.0	0.0 ± 2.6	1.0 ± 3.0	0.288	0.0 ± 1.1	1.0 ± 3.0	0.236
ct	1.0 ± 1.0	1.0 ± 1.0	1.0 ± 2.0	0.983	1.0 ± 1.0	1.0 ± 1.0	0.548
ci	1.0 ± 1.0	1.0 ± 1.0	1.0 ± 1.0	0.846	1.0 ± 1.0	1.0 ± 1.0	0.683
cv	2.0 ± 2.0	2.0 ± 2.0	2.0 ± 2.0	0.339	2.0 ± 3.0	2.0 ± 1.0	0.959
mm	1.0 ± 2.0	0.0 ± 1.8	1.0 ± 1.5	0.170	0.0 ± 1.8	1.0 ± 2.0	0.373
ah	3.0 ± 0.0	3.0 ± 0.0	3.0 ± 0.5	0.650	3.0 ± 0.4	3.0 ± 0.0	0.581
g	0.0 ± 1.0	0.0 ± 0.8	0.0 ± 1.8	0.589	0.0 ± 0.8	0.0 ± 1.5	0.581
t	0.0 ± 0.0	0.0 ± 0.0	0.0 ± 1.0	0.589	0.0 ± 0.0	0.0 ± 0.0	0.829
i	0.0 ± 0.0	0.0 ± 0.0	0.0 ± 1.0	0.156	0.0 ± 0.0	0.0 ± 0.0	0.905
v	0.0 ± 0.0	0.0 ± 0.0	0.0 ± 0.0	1.000	0.0 ± 0.0	0.0 ± 0.0	1.000
ptc	0.0 ± 0.0	0.0 ± 0.0	0.0 ± 2.0	0.170	0.0 ± 0.0	0.0 ± 1.0	0.256
C4d	0.0 ± 0.0	0.0 ± 0.0	0.0 ± 0.0	0.948	0.0 ± 0.0	0.0 ± 0.0	0.981
MVI sum score	0.0 ± 1.3	0.0 ± 0.8	0.0 ± 2.8	0.144	0.0 ± 0.8	0.0 ± 2.5	0.183
MVI sum score ≧2	7/30	1/16	6/13	**0.026**	1/12	6/15	0.091

Data were expressed as medians (interquartile range), or numbers

*BMI* body mass index, *BP* blood pressure, *eGFR* estimated glomerular filtration rate, *DSA* donor specific antibodies, *h/o* history of, *aTCMR* active T cell mediated rejection, *aABMR* active antibody-mediated rejection, *MVI* microvascular inflammation

**Fig. 1:**
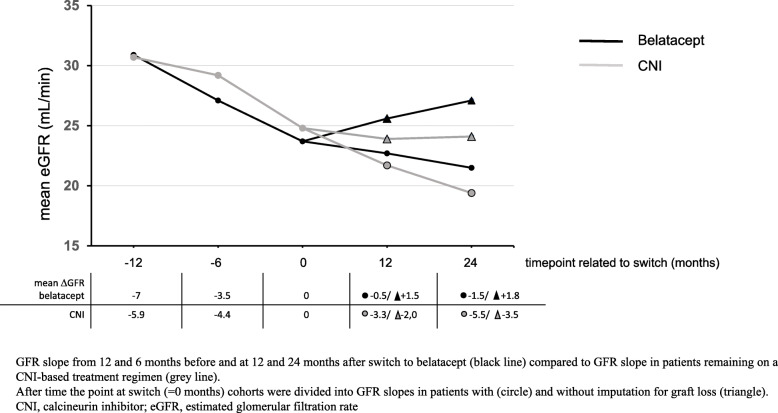
Mean eGFR slope before and after conversion

Median GFR at the time of conversion was 22.5 ± 12 (range 17–29.3) mL/min. In patients without graft loss GFR increased to 23.3 and 24.5 mL/min at 12 and 24 months, respectively. Median GFR with imputation for graft loss was 21.5 and 18.5 mL/min at 12/24 months. As depicted in Table 1 and Fig. 1, eGFR slopes at 12 and 6 months before switch to belatacept were ΔGFR of − 7.0 ± 8.2 and − 3.5 ± 6.3 mL/min., respectively. At 12 and 24 months after switch eGFR slope flattened in patients with imputation for graft loss (mean ΔGFR of − 0.56 ± 6.8 and − 1.54 ± 8.4 mL/min, respectively) and increased in patients without imputation for graft loss (mean ΔGFR of 1.3 ± 5.9 and 1.8 ± 7.9 mL/min).

14/30 (46.7%) patients were DSA-positive at the time of conversion. The median treatment duration on belatacept until graft loss, death or last follow-up was 29.5 ± 27 (range 11.8–38.8) months.

A kidney transplant biopsy was performed at a median time of 5 ± 11.3 (range 1–12.3) months prior to belatacept conversion in all patients. Rejection-related biopsy scores were assessed in detail (Tables 2 and 3). Seven patients displayed lesions showing a Banff microvascular inflammation (MVI) sum score of ≥2, one of these patients was DSA negative and did not display C4d + staining. Before conversion, 5/6 patients received specific treatment for active antibody-mediated rejection by plasmaphereses (*n* = 5/5), immunoglobulins (*n* = 3/5), bortezomib (*n* = 1/5), rituximab (*n* = 2/5), cyclophosphamide (*n* = 1/5) or thymoglobulin (*n* = 1/5).

### Patient survival at 12 and 24 months

Patient survival at 12 months was 96.7% (29/30), as one 60-year old patient died from sudden death at 8 months. Patient survival at 24 months was 90% (27/30), as one 67-year old patient died from central nervous system post-transplant lymphoproliferative disorder at month 22, and one 63-year old patient died from intracranial bleeding at month 13 after conversion.

### DSA, rejection and graft survival at 12 and 24 months

During 24 months follow-up, no de novo DSA were found. However, of 14 patients with DSA-positivity at the time of conversion, one became DSA-negative after 12 months, and another after 24 months. Interestingly, both had been identified as nonadherent by the treating physicians prior to belatacept conversion and showed treatment response after 12 and 24 months.

Only one overt rejection episode occurred in a 39-year old female combined pancreas and kidney recipient after belatacept conversion. This was a severe pancreas graft rejection, refractory to thymoglobulin and steroid bolus therapy, which lead to graft loss at 4 months during follow up. Only one kidney transplant biopsy was taken after conversion, showing no signs of acute humoral or cellular rejection.

Overall graft survival at 1 year was 76.7% (23/30) including one death with functioning graft, 4 kidney transplant recipients and the 2 combined pancreas and kidney transplant patients who experienced renal or pancreas graft losses, respectively (Table 1). Overall renal graft survival at 24 months was 60.0% (18/30) including 3 deaths with functioning grafts and 9 renal or pancreas graft losses, respectively. Graft survival censored for death was 79.3% (23/29) at 12 months and 66.7% (18/27) at 24 months (Table 1).

### Risk factors for renal graft loss at 12 and 24 months

In the univariate analysis of risk factors for graft loss, GFR < 25 mL/min (*p* = 0.042) and MVI sum score ≥ 2 (*p* = 0.023) at conversion were significant at 24 months (Table 2). In binary logistic regression analyses models including GFR < 25 mL/min at conversion and MVI sum score ≥ 2, only MVI sum score ≥ 2 was significant at 24 months (*p* = 0.038, OR 13.0, 95% CI 1.15–146.8, not shown).

Noteworthy, DSA-positivity at time of conversion was not associated with graft loss at 12 or 24 months.

### Risk factors for treatment failure at 12 and 24 months

In the univariate analysis of risk factors for treatment failure, only MVI sum score ≥ 2 was significant (*p* = 0.023) at 12 months (Table 3). In binary logistic regression analysis models including GFR < 25 mL/min at conversion and MVI sum score ≥ 2, only MVI sum score ≥ 2 was significant at 12 months (*p* = 0.037, OR 13.2, 95% CI 1.17–147.8, not shown).

Again, DSA-positivity was not associated with treatment failure at 12 or 24 months.

### Control cohort

#### Characteristics and outcome

We used a control cohort of 56 patients with CNI-based maintenance therapy to compare findings within the belatacept cohort for graft survival, outcome and renal function during the same observation period. Table 4 shows demographic and baseline characteristics of both cohorts. There were no significant differences e.g. regarding age, donor age, gender, time after transplantation, eGFR at switch, immunosuppression. Furthermore, except for arterial hyalinosis, no significant differences were observed regarding active and chronic lesions within renal transplant biopsies.

**Table 4:** Demographic and Clinical Characteristics of Belatacept and Control patients

Cohort characteristics	Belatacept (*N* = 30)	CNI (*N* = 56)	*P* value
Age (y)	53.5 ± 26	52.0 ± 21	0.700
Donor age (y)	48.0 ± 23	46.0 ± 23	0.907
Gender (m/f)	20/10	19/37	0.956
Post-transplant diabetes	3/30	5/56	0.871
BMI	25.3 ± 4.1	22.9 ± 8.3	0.213
Systolic BP (mmHg)	134 ± 17	130 ± 51	0.076
Diastolic BP (mmHg)	84 ± 10	80 ± 13	0.054
Time after transplantation (m)	127.5 ± 91.3	113.0 ± 102.5	0.500
eGFR (mL/min)	22.5 ± 12	24.5 ± 14	0.583
eGFR < 25 mL/min	17/30	28/56	0.667
Proteinuria (mg/g creatinine)	840 ± 1166	280.5 ± 1270	0.157
Living donor transplants	8/30	8/56	
Pancreas/kidney	2/30	6/56	
Immunosuppression			
Tacrolimus	22/30	42/56	0.867
Cyclosporine A	8/30	14/56	0.867
Mycophenolic acid	27/30	54/56	0.227
Azathioprin	2/30	0/56	0.052
Steroid	24/30	45/56	0.969
DSA	14/30	19/56	0.336
h/o any rejection	15/30	20/56	0.201
aTCMR	3/30	7/56	0.732
aABMR	6/30	12/56	0.990
Histology before switch (m)	5 ± 11.3	9.5 ± 26.5	0.236
Biopsy scores			
glomerular scarring (%)	30.5 ± 33	28.5 ± 45	0.942
cg	0.0 ± 3.0	0.0 ± 1.4	0.202
ct	1.0 ± 1.0	1.0 ± 1.0	1.000
ci	1.0 ± 1.0	1.0 ± 1.0	0.705
cv	2.0 ± 2.0	1.0 ± 1.0	0.368
mm	1.0 ± 2.0	1.0 ± 2.0	0.845
ah	3.0 ± 0.0	2.75 ±1.0	0.003
g	0.0 ± 1.0	0.0 ± 0.0	0.307
t	0.0 ± 0.0	0.0 ± 0.0	0.865
i	0.0 ± 0.0	0.0 ± 1.0	0.243
v	0.0 ± 0.0	0.0 ± 0.0	0.129
ptc	0.0 ± 0.0	0.0 ± 0.0	0.956
diffuse ptc (y/n)	0/30	3/56	0.333
C4d	0.0 ± 0.0	0.0 ± 0.0	1.000
MVI sum score	0.0 ± 1.3	0.0 ± 1.0	0.448
MVI sum score ≧2	7/30	8/56	0.295

Data were expressed as medians (interquartile range), or numbers

*BMI* body mass index, *BP* blood pressure, *eGFR* estimated glomerular filtration rate, *DSA* donor specific antibodies, *h/o* history of, *aTCMR* active T cell mediated rejection, *aABMR* active antibody-mediated rejection, *MVI* microvascular inflammation

Cases with DSA positivity (19 out of 56), and an MVI sum score ≥ 2 (8/56), were fewer in the control cohort compared to the belatacept cohort, while similar rates of active T-cell-mediated and active antibody-mediated rejections (aABMR) were observed in both groups. Eleven out of twelve aABMR received specific treatment prior to the start of observation time point (10 by plasmaphereses, 10 with immunoglobulins, 1 bortezomib, 7 with rituximab, 1 with cyclophosphamide). While eGFR slopes at 12 and 6 months prior to “virtual” switch were similar to eGFR slopes in belatacept patients (mean ΔGFR of − 5.9 ± 7.0 and − 4.4 ± 6.6 mL/min, respectively), eGFR slope did not flatten during 12 and 24 months follow up (mean ΔGFR of − 3.3 ± 7.6 and − 5.5 ± 8.3) in patients with imputation for graft loss. Moreover, in contrast to the belatacept group, eGFR did not increase in patients without imputation for graft loss at 12 and 24 months (mean ΔGFR of − 2.0 ± 7.2 and − 3.5 ± 8.6 mL/min, respectively). Results are shown in Table 1. Baseline characteristics by status for graft failure at 12 and 24 months censored for death are presented in the Additional file 1.

Notably, fewer patients in the control cohort had stable or improved renal function after 24 months (12/30 in belatacept patients versus 13/56 in control patients, Additional file 2).

## Discussion

Increasing numbers of studies reported the feasibility and safety of late switch to belatacept in certain patient groups, but still few is known on the benefit of belatacept-based treatment in patients with higher immunological risk for both the de novo and switch situations. Patient populations are often very heterogenous with different time points or treatment strategies before switch to belatacept. In our study we strictly focused on the switch of patients from CNI-based treatment to belatacept in the very late phase after transplantation (median of 10.6 ± 7.6 years).

The development of donor-specific antibodies (DSA) and subsequent antibody-mediated rejection (ABMR) are key factors for late allograft failure [19, 20]. While the percentage of patients with DSA in larger analyses was 20% by 5 years after transplantation [21], in our study a much higher percentage of 46.7% had DSA at the time of conversion.

However, we found no association of DSA positivity with graft loss or treatment failure at 12 or 24 months after conversion. Rather, we found an association of significant microvascular inflammation (MVI) score in biopsies prior to conversion to belatacept with treatment failure at 24 months, defined by glomerulitis (g) and peritubular capillaritis (ptc) ≥2, which is part of the histopathological criteria for diagnosis of active ABMR [17]. MVI has been validated in molecular studies and closely associated with outcome, even without C4d deposition or DSA [22–27].

Our data suggest that not DSA positivity per se, but the presence of active microvascular inflammation, which is suspicious, but not restricted to active humoral rejection, is a risk factor for patients that are converted from a CNI-based regimen to belatacept treatment.

Recently, a single center study on de novo belatacept use in a real life scenario including re-transplant patients, patients with higher panel reactive antibodies und HLA mismatches and higher percentages of “Afro American” patients found significantly increased acute rejection rates including more severe rejection grades with belatacept versus tacrolimus, ultimately leading to a modification of the immunosuppressive protocol including combined use of tacrolimus tapering until 9–12 months after transplantation [28].

Using thymoglobulin induction and belatacept in 49 patients with preformed DSA of mild mean fluorescence intensities (max 500–3000), Leibler et al. found no ABMR after 12 months, albeit again with significantly increased rates of T-cell-mediated rejections with an incidence of 25.4% [29].

In line with the previous phase II study by Rostaing et al., who converted patients between 6 and 36 months after kidney transplantation to belatacept, albeit with stable function, we included only patients with prolonged exposure to the calcineurin inhibitors tacrolimus or cyclosporine [12]. At 1 and 3 years, a significant improvement in kidney function compared with cyclosporine was found [9]. Acute rejection rates in belatacept-treated patients cumulated at 8.3% after 36 months [9]. Darres et al. described the results of conversion to belatacept in 219 kidney transplants including 35 (16%) patients with DSA and 9% patients with mTOR-based therapy from 5 European centers between 0 and 337 (mean 44) months after transplantation. Compared to our data, patients were converted earlier and displayed a higher eGFR at the time of conversion. Indication for conversion was mostly impaired kidney function but also intolerance to CNI or mTOR inhibitors. Graft loss occurred in 11% of patients at the end of follow up, and belatacept was stopped for other reasons in another 11% of patients. In the remaining cohort, eGFR increased from 32 to 38 mL/min, with the highest increase in patients switched before month 3 posttransplant. After conversion to belatacept 8.2% of patients developed an acute rejection episode, and 3 patients developed DSA. The authors concluded that overall efficacy and safety were good, even in patients with DSA [14].

In our study, DSA positivity was not associated with graft or treatment failure at 12 and 24 months, only one overt pancreas graft rejection (3.3%) occurred leading to graft loss at 4 months after conversion. Interestingly, no patient developed de novo DSA after belatacept conversion.

One limit of our study is the lack of follow-up biopsies on patients with graft deterioration, as only one follow-up biopsy was performed, showing no rejection.

Dürr et al. reported the conversion of 69 renal transplant patients including 20% patients under mTOR inhibitors to belatacept at a mean time of 68.8 months after transplantation. 38% showed a significant eGFR increase after 12 months [15]. Notably, DSA mean fluorescence intensities after conversion as well as higher proteinuria before conversion associated with non-responder status after 12 months. In contrast to the present study, higher proteinuria at the time of conversion was associated with less GFR increase. This might be related to exclusion of patients with mTOR-inhibitor-based treatment in our present study.

Notably, in our study both patients with combined pancreas and renal grafts experienced graft losses during 24 months follow up. Beside the pancreas graft loss due to rejection, the other patient lossed his kidney graft due to unknown reasons. Few data are available on switching pancreas transplant patients to belatacept. A previous study reported successful conversion of two patients from tacrolimus to belatacept and sirolimus [30]. Furthermore, belatacept together with sirolimus has been successfully used in rhesus monkeys with islet transplantation [31]. Thus, more data are needed to evaluate safety and efficacy in these patients.

To draw stronger conclusions about the results of our study, we compared the belatacept group with a control cohort under CNI maintenance therapy. We could demonstrate comparable outcome results regarding death and graft survival, albeit no better outcome regarding renal function. Compared to controls, patients in the belatacept group slowed loss of renal function after conversion during 12 and 24 months follow up and moreover increased GFR in the analysis in cases without imputation for graft loss. However, due to the retrospective nature of our study, data are biased and interpretation of differences between both groups has to be done cautiously. An ongoing large prospective, randomized trial will provide more robust data on the outcome of maintenance kidney transplant recipients following conversion to belatacept (NCT 01820572).

The limitations of our study are the small sample size limiting significant findings, albeit we included a high proportion of patients at immunological risk. The follow-up time for our study was still short, and belatacept may be more effective in preserving eGFR beyond 2 years after conversion. But due to a very late belatacept switch after transplantation with a higher risk of terminal transplant failure longer follow up might be difficult to interpret.

## Conclusions

Rescue therapy with conversion from CNI to belatacept is feasible in patients with worsening renal function, even many years after transplantation and in DSA-positive patients. However, patients at immunological risk such as those with combined pancreas and renal transplants, history of rejection, significant microvascular inflammation in biopsy and patients with severe graft impairment should be treated with caution and have a more in-depth evaluation in controlled studies to define the benefit of a conversion to belatacept to avoid rejection and graft losses.

## Supplementary information

**Additional file 1.** Baseline characteristics of Control cohort by status for graft failure at 12 and 24 months censored for death.

**Additional file 2.** Baseline characteristics of Control cohort by status for treatment success or failure censored for death.

## Data Availability

The data generated and used in the analysis of this study are included in this published article. Additional data is available from the authors upon reasonable request.

## Abbreviations

aABMR: Active antibody-mediated rejection
aTCMR: Active T-cell-mediated rejection
BMI: Body mass index
CNI: Calcineurin inhibitor
DSA: Donor-specific antibody
GFR: Glomerular filtration rate
KTR: Kidney transplant recipient
mTOR: Mammalian target of rapamycin
MVI: Banff microvascular inflammation

## References

[1] Nankivell BJ, Borrows RJ, Fung CL, O’Connell PJ, Allen RD, Chapman JR (2003). The natural history of chronic allograft nephropathy. N Engl J Med.

[2] Gaston RS (2009). Chronic calcineurin inhibitor nephrotoxicity: reflections on an evolving paradigm. Clin J Am Soc Nephrol.

[3] Vincenti F, Larsen CP, Alberu J, Bresnahan B, Garcia VD, Kothari J (2012). Three-year outcomes from BENEFIT, a randomized, active-controlled, parallel-group study in adult kidney transplant recipients. Am J Transplant.

[4] Vincenti F (2016). Belatacept and long-term outcomes in kidney transplantation. N Engl J Med.

[5] Durrbach A, Pestana JM, Florman S, Del Carmen RM, Rostaing L, Kuypers D (2016). Long-term outcomes in Belatacept- versus cyclosporine-treated recipients of extended criteria donor kidneys: final results from BENEFIT-EXT, a phase III randomized study. Am J Transplant.

[6] Vincenti F, Blancho G, Durrbach A, Grannas G, Grinyo J, Meier-Kriesche HU (2017). Ten-year outcomes in a randomized phase II study of kidney transplant recipients administered belatacept 4-weekly or 8-weekly. Am J Transplant.

[7] Leibler C, Matignon M, Pilon C, Montespan F, Bigot J, Lang P (2014). Kidney transplant recipients treated with belatacept exhibit increased naive and transitional B cells. Am J Transplant.

[8] Leibler C, Thiolat A, Henique C, Samson C, Pilon C, Tamagne M (2018). Control of humoral response in renal transplantation by Belatacept depends on a direct effect on B cells and impaired T follicular helper-B cell crosstalk. J Am Soc Nephrol.

[9] Grinyo JM, Del Carmen RM, Alberu J, Steinberg SM, Manfro RC, Nainan G (2017). Safety and efficacy outcomes 3 years after switching to Belatacept from a Calcineurin inhibitor in kidney transplant recipients: results from a phase 2 randomized trial. Am J Kidney Dis.

[10] Nair V, Liriano-Ward L, Kent R, Huprikar S, Rana M, Florman SS (2017). Early conversion to belatacept after renal transplantation. Clin Transpl.

[11] Le Meur Y, Aulagnon F, Bertrand D, Heng AE, Lavaud S, Caillard S (2016). Effect of an early switch to Belatacept among Calcineurin inhibitor-intolerant graft recipients of kidneys from extended-criteria donors. Am J Transplant.

[12] Rostaing L, Massari P, Garcia VD, Mancilla-Urrea E, Nainan G, del Carmen RM (2011). Switching from calcineurin inhibitor-based regimens to a belatacept-based regimen in renal transplant recipients: a randomized phase II study. Clin J Am Soc Nephrol.

[13] Brakemeier S, Kannenkeril D, Durr M, Braun T, Bachmann F, Schmidt D (2016). Experience with belatacept rescue therapy in kidney transplant recipients. Transpl Int.

[14] Darres A, Ulloa C, Brakemeier S, Garrouste C, Bestard O, Del Bello A (2018). Conversion to Belatacept in maintenance kidney transplant patients: a retrospective multicenter European study. Transplantation.

[15] Durr M, Lachmann N, Zukunft B, Schmidt D, Budde K, Brakemeier S (2017). Late conversion to Belatacept after kidney transplantation: outcome and prognostic factors. Transplant Proc.

[16] Gupta S, Rosales I, Wojciechowski D (2018). Pilot analysis of late conversion to Belatacept in kidney transplant recipients for biopsy-proven chronic tacrolimus toxicity. J Transp Secur.

[17] Loupy A, Haas M, Solez K, Racusen L, Glotz D, Seron D (2017). The Banff 2015 kidney meeting report: current challenges in rejection classification and prospects for adopting molecular pathology. Am J Transplant.

[18] Fritsche L, Schroter K, Lindemann G, Kunz R, Budde K, Neumayer HH (1933). A web-based electronic patient record system as a means for collection of clinical data. Lect Notes Comput Sc.

[19] Einecke G, Sis B, Reeve J, Mengel M, Campbell PM, Hidalgo LG (2009). Antibody-mediated microcirculation injury is the major cause of late kidney transplant failure. Am J Transplant.

[20] Sellares J, de Freitas DG, Mengel M, Reeve J, Einecke G, Sis B (2012). Understanding the causes of kidney transplant failure: the dominant role of antibody-mediated rejection and nonadherence. Am J Transplant.

[21] Everly MJ, Rebellato LM, Haisch CE, Ozawa M, Parker K, Briley KP (2013). Incidence and impact of de novo donor-specific alloantibody in primary renal allografts. Transplantation.

[22] de Kort H, Willicombe M, Brookes P, Dominy KM, Santos-Nunez E, Galliford JW (2013). Microcirculation inflammation associates with outcome in renal transplant patients with de novo donor-specific antibodies. Am J Transplant.

[23] Gupta A, Broin PO, Bao Y, Pullman J, Kamal L, Ajaimy M (2016). Clinical and molecular significance of microvascular inflammation in transplant kidney biopsies. Kidney Int.

[24] Kozakowski N, Herkner H, Bohmig GA, Regele H, Kornauth C, Bond G (2015). The diffuse extent of peritubular capillaritis in renal allograft rejection is an independent risk factor for graft loss. Kidney Int.

[25] Kozakowski N, Herkner H, Eskandary F, Eder M, Winnicki W, Klager J (2019). An integrative approach for the assessment of peritubular capillaritis extent and score in low-grade microvascular inflammation-associations with transplant glomerulopathy and graft loss. Nephrol Dial Transplant.

[26] Loupy A, Hill GS, Suberbielle C, Charron D, Anglicheau D, Zuber J (2011). Significance of C4d Banff scores in early protocol biopsies of kidney transplant recipients with preformed donor-specific antibodies (DSA). Am J Transplant.

[27] Sis B, Jhangri GS, Riopel J, Chang J, de Freitas DG, Hidalgo L (2012). A new diagnostic algorithm for antibody-mediated microcirculation inflammation in kidney transplants. Am J Transplant.

[28] Adams AB, Goldstein J, Garrett C, Zhang R, Patzer RE, Newell KA (2017). Belatacept combined with transient Calcineurin inhibitor therapy prevents rejection and promotes improved long-term renal allograft function. Am J Transplant.

[29] Leibler C, Matignon M, Moktefi A, Samson C, Zarour A, Malard S (2019). Belatacept in renal transplant recipient with mild immunologic risk factor: a pilot prospective study (BELACOR). Am J Transplant.

[30] Mujtaba MA, Sharfuddin AA, Taber T, Chen J, Phillips CL, Goble M (2014). Conversion from tacrolimus to belatacept to prevent the progression of chronic kidney disease in pancreas transplantation: case report of two patients. Am J Transplant.

[31] Lowe MC, Badell IR, Turner AP, Thompson PW, Leopardi FV, Strobert EA (2013). Belatacept and sirolimus prolong nonhuman primate islet allograft survival: adverse consequences of concomitant alefacept therapy. Am J Transplant.

