# Supplementary material for: Microvascular inflammation is a risk factor in kidney transplant recipients with very late conversion from calcineurin inhibitor-based regimens to belatacept
Source: BMC Nephrol. 2020 Aug 20;21:354. doi: 10.1186/s12882-020-01992-6 (PMC7439694; doi:10.1186/s12882-020-01992-6)
Supplement: Supplementary file 1 — Additional file 1. Baseline characteristics of Control cohort by status for graft failure at 12 and 24 months censored for death. [file 12882_2020_1992_MOESM1_ESM.doc]

**Additional file 1. Baseline characteristics of Control cohort** by status for graft failure at 12 and 24 months censored for death.

| Patient  characteristics | All  patients  (N=56) | Functioning graft  at 12 months  (N=46) | Graft failure  at 12 months  (N=8) | Functioning graft  at 24 months  (N=35) | Graft failure  at 24 months  (N=16) |
| --- | --- | --- | --- | --- | --- |
| Age (y) | 52.0 ±21 | 53.5 ±21 | 46.5 ±17 | 53.0 ±23 | 49 ±12 |
| Donor age (y) | 46.0 ±23 | 47.0 ±24 | 42.5 ±25 | 44.5 ±25 | 44.0 ±25 |
| Gender (m/f) | 19/37 | 31/15 | 4/4 | 25/10 | 8/8 |
| Post-transplant diabetes | 5/56 | 5/46 | 0/8 | 5/35 | 0/16 |
| BMI | 22.9 ±8.3 | 23.4 ±8.2 | 21.5 ±8.1 | 23.2 ±8.7 | 22.7 ±7.3 |
| Systolic BP (mmHg)  Diastolic BP (mmHg) | 134 ±17  84 ±10 | 130.5 ±19  79.5 ±14 | 137 ±34  81 ±16 | 130 ±19  79 ±14 | 130.5 ±28  80 ±10 |
| Time after transplantation (m) | 113 ±102.5 | 115 ±104.8 | 112.5 ±156.8 | 114 ±104 | 137 ±158 |
| eGFR (mL/min) | 24.5 ±14 | 27.0 ±14 | 20.5 ±10 | 29.0 ±11 | 19.5 ±10 |
| eGFR < 25 mL/min | 28/56 | 19/46 | 7/8 | 10/35 | 14/16 |
| Proteinuria (mg/g creatinine) | 260 ±1166 | 255 ±1276 | 937 ±1676 | 223 ±591 | 937 ±1900 |
| Living donor transplants  pancreas/kidney | 8/56  6/56 | 7/46  5/46 | 1/8  0/46 | 5/35  4/35 | 3/16  1/46 |
| Immunosuppression  Tacrolimus  Cyclosporine A  Mycophenolic acid  Azathioprin  Steroid | 42/56  14/56  54/56  0/56  45/56 | 33/46  13/46  45/46  0/46  35/46 | 7/8  1/8  7/8  0/8  8/8 | 25/35  10/35  35/35  0/35  25/35 | 14/16  2/16  14/16  0/16  15/16 |
| DSA  h/o any rejection  aTCMR  aABMR | 19/56  20/56  7/56  12/56 | 14/46  16/46  6/46  11/46 | 4/8  3/8  1/8  2/8 | 9/35  12/35  3/35  6/35 | 8/16  7/16  3/16  6/16 |

Data were expressed as medians (interquartile range), or numbers

BMI body mass index, BP blood pressure, eGFR estimated glomerular filtration rate, DSA donor specific antibodies, h/o history of, aTCMR active T cell mediated rejection, aABMR, active antibody-mediated rejection
