# Supplementary material for: Microvascular inflammation is a risk factor in kidney transplant recipients with very late conversion from calcineurin inhibitor-based regimens to belatacept
Source: BMC Nephrol. 2020 Aug 20;21:354. doi: 10.1186/s12882-020-01992-6 (PMC7439694; doi:10.1186/s12882-020-01992-6)
Supplement: Supplementary file 2 — Additional file 2. Baseline characteristics of Control cohort by status for treatment success or failure censored for death. [file 12882_2020_1992_MOESM2_ESM.doc]

**Additional file 2. Baseline characteristics of Control cohort by status for treatment success or failure censored for death**.

Treatment failure was defined as graft failure or GFR deterioration compared to the time of conversion

| Patient  characteristics | All  patients  (N=56) | Treatment success  at 12 months  (N=17) | Treatment failure  at 12 months  (N=37) | Treatment success  at 24 months  (N=13) | Treatment failure  at 24 months  (N=38) |
| --- | --- | --- | --- | --- | --- |
| Age (y) | 52.0 ±21 | 58.0 ±19 | 49 ±19 | 54.0 ±19 | 51.0 ±23 |
| Donor age (y) | 46.0 ±23 | 46.0 ±32 | 48 ±23 | 42.0 ±25 | 47 ±25 |
| Gender (m/f) | 19/37 | 13/4 | 22/15 | 10/3 | 23/15 |
| Post-transplant diabetes | 5/56 | 3/17 | 2/37 | 3/13 | 2/38 |
| BMI | 22.9 ±8.3 | 22.9 ±7.8 | 22.9 ±8.4 | 23.6 ±7.1 | 22.9 ±8.6 |
| Systolic BP (mmHg)  Diastolic BP (mmHg) | 134 ±17  84 ±10 | 131 ±18  77 ±15 | 130 ±21  80 ±12 | 130.0 ±17  80 ±13 | 131 ±20  79.5 ±13 |
| Time after transplantation (m) | 113 ±102.5 | 116 ± 84.4 | 114 ±83.7 | 88 ±56.4 | 124 ±88.4 |
| eGFR (mL/min) | 24.5 ±14 | 25.0 ±13 | 25.0 ±15 | 25.0 ±12 | 25.0 ±14 |
| eGFR < 25 mL/min | 28/56 | 8/17 | 18/37 | 6/13 | 18/38 |
| Proteinuria (mg/g creatinine) | 260 ±1166 | 124 ±117 | 534 ±1516 | 124 ±136 | 471 ±1504 |
| living donor transplants  pancreas/kidney | 8/56  6/56 | 3/17  - | 5/37  - | 3/13 | 5/38 |
| Immunosuppression  Tacrolimus  Cyclosporine A  Mycophenolic acid  Azathioprin  Steroid | 42/56  14/56  54/56  0/56  45/56 | 10/17  7/17  17/17  0/17  14/17 | 30/37  7/37  35/37  0/37  29/37 | 8/13  5/13  13/13  0/13  10/13 | 31/38  7/38  36/38  0/38  30/38 |
| DSA  h/o any rejection  aTCMR  ABMR | 19/56  20/36  7/56  13/56 | 5/15  5/17  1/17  3/17 | 13/36  14/37  6/37  10/37 | 0/11  5/13  1/13  2/13 | 17/37  14/38  5/38  10/15 |

Data were expressed as medians (interquartile range), or numbers

BMI body mass index, BP blood pressure, eGFR estimated glomerular filtration rate, DSA donor specific antibodies, h/o history of, aTCMR active T cell mediated rejection, aABMR, active antibody-mediated rejection
